# Consumption of sugar-sweetened beverages and fast foods deteriorates adolescents' mental health

**DOI:** 10.3389/fnut.2022.1058190

**Published:** 2022-12-22

**Authors:** Jin Suk Ra

**Affiliations:** College of Nursing, Chungnam National University, Daejeon, South Korea

**Keywords:** adolescent, sugar-sweetened beverages, fast foods, suicidal ideation, psychological stress, depression

## Abstract

**Introduction:**

Sugar-sweetened beverage (SSB) and fast-food consumption is significantly associated with adolescents' poor mental health. Furthermore, sugar-sweetened beverage and fast-food consumption might form clustered diet patterns with significant positive associations in adolescent high school students. Thus, the combined consumption of SSBs and fast foods may have more negative effects on mental health with synergetic effects than the sum of their independent consumption.

**Methods:**

This study aimed to identify the effects of combining the consumption of sugar-sweetened beverages and fast foods on mental health, including stress, depressive symptoms, and suicidal ideation among Korean high school students. Secondary data from 24,006 high school students were analyzed from the 17th Korea Youth Risk Behavior Web-based Survey, 2021. For statistical analysis, complex sampling analysis using the SPSS Statistics 26.0 software was applied for descriptive statistics and logistic regression analysis.

**Results:**

In Korean adolescents, combining more than medium consumption of sugar-sweetened beverages and fast foods was associated with more stress, depressive symptoms, and suicidal ideation than their independent consumption. In addition, combining high consumption of sugar-sweetened beverages and low to high consumption of fast foods might have dose-dependent negative effects on stress, depressive symptoms, and suicidal ideation in Korean adolescents.

**Discussion:**

Based on the results of this study, healthcare providers in schools and communities might develop various interventions including school/community-based feeding programs and policies targeting the restriction of SSB and fast-food consumption to improve adolescents' mental health.

## Introduction

Adolescence is considered a significant period for establishing independent diet patterns from parents that last for a lifetime (1). Adolescents in high school spend the day at school, where they might be prone to consuming sugar-sweetened beverages (SSBs, e.g., soda, fruit-flavored drinks, sports drinks) and fast foods (e.g., hamburgers, pizza) *via* cafeterias and vending machines (2). In addition, high school students tend to eat at fast-food restaurants with their friends as a convenient place to spend their leisure time (3). Studies have reported that 63% of adolescents in the USA consumed SSBs at least once a day, while approximately 94% of Korean high school students consumed SSBs at least once a week, and approximately 40% of them consumed SSBs more than once a day (4, 5). In addition, according to a global school-based survey, adolescents consumed fast foods an average of 1.05 times a week (6). Based on national data in 2019, 82.5% of Korean adolescents consumed fast foods more than once a week (7). Thus, frequent SSB and fast-food consumption is a widespread diet pattern among adolescents (2, 8).

Regarding physical health, frequent SSB and fast-food consumption was significantly associated with developing obesity and metabolic syndromes, such as type 2 diabetes and dyslipidemia (9–11). In addition, SSB and fast-food consumption were associated with adolescents' poor mental health, including stress, depression, and suicidality (12–15). Adolescence is a developmental period with more mental health problems than in other developmental periods. Thus, dietary behaviors that are potentially associated with mental health are important in preventing mental health problems in adolescents (12, 16). Moreover, Korean high school students may have poor mental health with decreased resilience against stressors due to increased academic competition and a social climate that demands higher academic performance (17). In this context, reducing the consumption of SSBs and fast foods as unhealthy diet behaviors may be important for improving Korean adolescents' mental health. However, in a previous study of adolescents from 32 countries, the significance of the association between SSB and fast-food consumption and mental health varied (13). Liu et al. (18) proposed that the inconsistent findings were associated with ethnicity and the definitions of consumption of diet and mental health. Thus, the association between SSB and fast-food consumption and mental health should be evaluated in Korean adolescents.

Furthermore, SSB and fast-food consumption might form clustered diet patterns with significant positive associations in adolescent high school students (2, 19). According to Kang et al. (20), combining lifestyle behaviors may present neutralized or synergistic effects on health. Thus, the combined consumption of SSBs and fast foods may have more negative effects on mental health with synergetic effects than the sum of their independent consumption. Thus, hypothesis of this study was that combined consumption of SSBs and fast foods would have more negative effects on mental health of adolescent high school students than their independent consumption. However, considering the multi-dimensional effects of covariates, identifying the effects of the combined consumption of SSBs and fast foods on mental health in adolescents was limited. Nevertheless, the significance of their effects on mental health differed according to controlled covariates in adolescents (21, 22).

According to the biopsychosocial model proposed by Engel (23), individuals' health was influenced by biological (e.g., age, sex), social (e.g., socioeconomic status of the family), and psychological factors (e.g., mood, health behaviors). The biopsychosocial model is an appropriate framework for understanding the interactive influence of these three factors on individual health. It encourages a comprehensive understanding of individuals' unique characteristics associated with their health statuses (23, 24). In the literature review, as covariates, associated factors of adolescents' mental health were: sex (25) and sleep satisfaction (25) in biological factors; grade (25), academic achievement (25), living with family members (25), family's socioeconomic status (25), type of school (25), and area of residential location in social factors (25); and perceived health status (26), perceived body shape (27), skipping breakfast (28), screen-based sedentary time (29), moderate and vigorous physical activity (30), current smoking consumption (25), current alcohol consumption (25), the experience of sexual intercourse (31), and experience of substance use (25) in psychological factors. Thus, this study aimed to identify the effects of combining the consumption of SSBs and fast foods on mental health, including stress, depressive symptoms, and suicidal ideation of Korean high school students, who participated in the 17th Korea Youth Risk Behavior Web-based Survey (KYRBS), after controlling for covariates.

## Materials and methods

### 2.1 Research design and sample

Using a cross-sectional study design, secondary data analysis was performed on the data obtained from the 17^*th*^ KYRBS, 2021, a national survey of middle and high school students. A total of 59,426 students were recruited from 800 schools across 17 provinces of South Korea, and 54,848 adolescents (92.9%) participated in the survey. As inclusion criteria of the sample, high school students who answered questions regarding outcome, independent variables, and covariates were included Thus, data from 24,006 high school students who answered questions on mental health (stress, depressive symptoms, and suicidal ideation); SSB and fast-food consumption; and covariates (biological, social, and psychosocial factors potentially associated with mental health) were analyzed in this study.

### 2.2 Measurements

#### 2.2.1 Outcome variables

##### 2.2.1.1 Stress

Stress was evaluated with a single item assessing participants' usual experience of stress levels. The response was classified into “with stress” (including *not so much, a little, often*, and *very often*) and “without stress” (including *not at all*).

##### 2.2.1.2 Depressive symptoms

Depressive symptoms were evaluated with a single item assessing participants' experience of a sense of sadness or hopelessness in the last 12 months. The response was classified into “with depressive symptoms” or “without depressive symptoms.”

##### 2.2.1.3 Suicidal ideation

Suicidal ideation was evaluated with a single item assessing whether participants seriously considered suicide within the last 12 months. The response was classified into “with suicidal ideation” or “without suicidal ideation.”

#### 2.2.2 Independent variables

##### 2.2.2.1 SSB consumption

SSB consumption was assessed using two items evaluating daily consumption of soda and other sweetened drinks in the last seven days. According to the response scale, the frequency of SSB consumption was converted into times per week (e.g., 3–4 times a week = 3.5 times a week, once daily = 7 times a week) for each soda and other sweetened drinks, respectively. Thereafter, the frequency of SSB consumption was calculated by adding the converted values (times per week) for each soda and other sweetened drinks. Finally, the frequency of SSB consumption was classified into three quartile groups (first quartile (Q1) = low consumption, second quartile (Q2) = medium consumption, third quartile (Q3) = high consumption).

##### 2.2.2.2 Fast-food consumption

Fast-food consumption was assessed using a single item assessing participants' daily consumption of fast foods in the last seven days. According to the response scale, the frequency of fast-food consumption was converted into times per week (e.g., 3–4 times a week = 3.5 times a week, once daily = 7 times a week). Finally, the frequency of fast-food consumption was classified into three quartile groups (first quartile (Q1) = low consumption, second quartile (Q2) = medium consumption, third quartile (Q3) = high consumption).

#### 2.2.3 Covariates

The questions used to assess biological, social, and psychological factors of covariates and their responses are given in Table 1.

**Table 1:** Measurement of covariates.

**Variables**	**Measurement**
**Biological factors**	
Sex	Boys or girls
Sleep satisfaction	Assessed with a single question regarding sleep satisfaction in the last 7 days. Responses were classified into dissatisfied (very dissatisfied and dissatisfied) or satisfied (very satisfied and satisfied)
**Social factors**	
Grade	1st, 2nd, or 3rd
Academic achievement	Assessed with a single question regarding perceived academic achievement. Response were classified into high, middle (including upper-middle, middle, lower-middle), or low
Living with family members	Assessed with a single question regarding living with family members. Response were classified into yes or no (living with others, but family)
Family's socioeconomic status	Assessed with a single question regarding perceived socioeconomic status of family. Response were classified into high, middle (including upper-middle, middle, lower-middle), or low
Type of school	Specialized high school or general high school
Area of residential location	Metropolis, middle-sized city, or rural area
**Psychological factors**	
Perceived health status	Assessed with a single question regarding perceived health status. Responses were classified into healthy (very healthy and healthy), fair, or unhealthy (very unhealthy and unhealthy)
Perceived body shape	Assessed with a single question regarding perceived body shape. Responses were classified into being fat (very and slightly fat), in average (not fat and not skinny), or skinny (very and slightly skinny)
Skipping breakfast	Assessed with a single question regarding days of having breakfasts in the last 7 days. Responses were classified into yes (0–6 days of having breakfast with days of skipping breakfast) or no (7 days of having breakfast without days of skipping breakfast)
Screen-based sedentary time	Assessed with a single question regarding average hours or minutes, a day with screen-based sedentary activities (watching TV, playing video games, using the internet, except for learning purpose). Responses were classified into ≥2 h or <2 h a day
Moderate and vigorous physical activity	Assessed with two questions regarding number of days for moderate and vigorous physical activity in the last 7 days. Responses were classified into ≥ 3 days or <3 days a day based on the physical activity guideline for Korean, 2013
Current smoking consumption	Assessed with a single question regarding smoking consumption experiences (days) in the last 30 days. Responses were classified into yes (≥1–2 days) or no (never)
Current alcohol consumption	Assessed with a single question regarding alcohol consumption experiences (days) in the last 30 days. Responses were classified into yes (≥1–2 days) or no (never)
Experience of sexual intercourse	Assessed with a single question regarding experience of sexual intercourse. Available responses were yes or no
Experience of substance use	Assessed with a single question regarding habitual use of substance except for therapeutic purpose. Available responses were yes or no

### 2.3 Ethical considerations

Since a secondary data analysis was conducted using the 17^*th*^ KYRBS, 2021, this study was exempted from the Institutional Review Board's (IRB) review (Approval no. 202209-SB-121-01).

### 2.4 Statistical analysis

Following the guidelines proposed by the 17^*th*^ KYRBS, 2021, a complex sampling analysis was performed using SPSS, version 26.0 (IBM, Armonk, NY, USA). As a first step of the complex sampling analysis, an analysis plan file was created with adjusting strata, clustering, and weight of samples. Then, descriptive and logistic analysis in the complex sampling analysis was conducted with the analysis plane file. The descriptive statistics were applied to analyze the prevalence (frequency and percentage) of stress, depressive symptoms, suicidal ideation, SSB and fast-food consumption, and covariates. To identify the effects of combining consumption of SSBs and fast foods on stress, depressive symptoms, and suicidal ideation, the logistic regression analysis in the complex analysis was applied.

## Results

### 3.1 Prevalence of stress, depressive symptoms, suicidal ideation, and SSB and fast-food consumption

Of the participants, 82.5% reported that they had experienced stress, while 27.4% had experienced depressive symptoms (sense of sadness or hopelessness) in the last 12 months. Moreover, 11.9% reported having suicidal ideation during the last 12 months (Table 2).

**Table 2:** Prevalence of stress, depressive symptoms, suicidal ideation, and sugar-sweetened beverage and fast-food consumption (*N* = 24,006).

**Variables**	**Categories**	***n* (%)**
**Mental health**		
Stress	With	19,806 (82.5)
	Without	4,200 (17.5)
Depressive symptoms	With	6,579 (27.4)
	Without	17,427 (72.6)
Suicidal ideation	With	2,852 (11.9)
	Without	21,154 (88.1)
**Independent consumption of sugar-sweetened beverages and fast-foods**		
Sugar-sweetened beverage consumption	Q1 (low)	8,917 (37.1)
	Q2 (medium)	9,855 (41.1)
	Q3 (high)	5,234 (21.8)
Fast-food consumption	Q1 (low)	17,301 (71.8)
	Q2 (medium)	5,321 (22.3)
	Q3 (high)	1,384 (5.9)
**Combining consumption of sugar-sweetened beverages and fast-foods**		
Sugar-sweetened beverage consumption	Fast-food consumption	
Q1 (low)	Q1 (low)	7,553 (31.5)
	Q2 (medium)	1,225 (5.1)
	Q3 (high)	139 (0.6)
Q2 (medium)	Q1 (low)	6,993 (29.1)
	Q2 (medium)	2,420 (10.1)
	Q3 (high)	442 (1.8)
Q3 (high)	Q1 (low)	2,755 (11.5)
	Q2 (medium)	1,676 (7.0)
	Q3 (high)	803 (3.3)
**Covariates**		
Biological factors		
Sex	Boys	12,441 (51.9)
	Girls	11,565 (48.1)
Sleep satisfaction	Dissatisfied	12,137 (50.6)
	Satisfied	11,869 (49.4)
**Social factors**		
Grade	1st	8,175 (31.8)
	2nd	8,348 (33.8)
	3rd	7,483 (34.4)
Academic achievement	Low	8,915 (36.9)
	Middle	7,839 (32.8)
	High	7,252 (30.3)
Living with family members	Yes	1,753 (6.4)
	No	22,253 (93.6)
Family's socioeconomic status	Low	3,430 (13.8)
	Middle	12,411 (51.3)
	High	8,165 (34.9)
Type of school	Specialized high school	4,255 (16.5)
	General high school	19,751 (83.5)
Area of residential location	Metropolis	12,110 (50.5)
	Middle sized city	10,430 (45.1)
	Rural area	1,466 (4.4)
**Psychological factors**		
Perceived health status	Unhealthy	2,563 (10.7)
	Fair	6,372 (26.5)
	Healthy	15,071 (62.8)
Perceived body shape	Being fat	9,661 (39.8)
	In average	8,575 (35.8)
	Skinny	5,770 (24.4)
Skipping breakfast	Yes	13,280 (55.4)
	No	10,726 (44.6)
Screen-based sedentary time (a day)	≥2 h	19,530 (81.5)
	<2 h	4,476 (18.5)
Moderate and vigorous physical activity (a week)	≥3 days	8,396 (34.5)
	<3 days	15,610 (65.5)
Current smoking consumption	Yes	1,985 (8.1)
	No	22,021 (91.9)
Current alcohol consumption	Yes	3,864 (15.9)
	No	20,142 (84.1)
Experience of sexual intercourse	Yes	2,026 (8.4)
	No	21,980 (91.6)
Experience of substance use	Yes	195 (0.8)
	No	23,811 (99.2)

n = unweighted, % = weighted.

Q1, first quantile; Q2, second quantile; Q3, third quantile.

When the level of SSB and fast-food consumption was independently classified into Q1 (low)–Q3 (high), 37.1, 41.1, and 21.8% of participants fell into Q1, Q2, and Q3, respectively. In addition, 71.8, 22.3, and 5.9% of participants fell into Q1, Q2, and Q3, respectively (Table 2).

Regarding combining SSB and fast-food consumption, 31.5% of the participants were in Q1 of SSB and fast-food consumption. In addition, 5.1% were in Q1 of SSB consumption and Q2 of fast-food consumption, and 0.6% were in Q1 of SSB consumption and Q3 of fast-food consumption. In addition, 29.1% of participants were in Q2 of SSB consumption and Q1 of fast-food consumption, 10.1% were in Q2 of SSB consumption and Q2 of fast-food consumption, and 1.8% were in Q2 of SSB consumption and Q3 of fast-food consumption. Finally, 11.5% of participants were in Q3 of SSB consumption and Q1 of fast-food consumption, 7.0% were in Q3 of SSB consumption and Q2 of fast-food consumption, and 3.3% were in Q3 of SSB consumption and Q3 of fast-food consumption (Table 2).

### 3.2 Effects of independent consumption of SSBs and fast foods on mental health in adolescents

Compared to Q1 of SSB consumption (reference), Q2 (Adjusted odds ratio [AOR] = 1.07, 95% confidence interval [CI] = 1.00–1.14) and Q3 of SSB consumption were associated with increased stress (AOR = 1.20, 95% CI = 1.11–1.29). In addition, Q3 of SSB consumption was associated with increased depressive symptoms (AOR = 1.19, 95% CI = 1.09–1.30) and suicidal ideation (AOR = 1.18, 95% CI = 1.05–1.32) compared to the reference (Table 3).

**Table 3:** Effects of independent consumption of sugar-sweetened beverages and fast-foods on mental health in adolescents (*N* = 24,006).

**Unhealthy lifestyle factors**		**Stress**	**Depressive symptoms**	**Suicidal ideation**
		**Adjusted odds ratio (95% confidence interval)** ^†^		
Sugar-sweetened beverage consumption	Q1 (low)	1.00	1.00	1.00
	Q2 (medium)	1.07 (1.00–1.14)^*^	1.05 (0.98–1.13)	1.02 (0.92–1.21)
	Q3 (high)	1.20 (1.11-1.29)^*^	1.19 (1.09–1.30)^*^	1.18 (1.05–1.32)^*^
Fast-food consumption	Q1 (low)	1.00	1.00	1.00
	Q2 (medium)	1.05 (0.99–1.12)	1.16 (1.08–1.25)^*^	0.99 (0.90–1.09)
	Q3 (high)	1.11 (0.99–1.25)	1.38 (1.21–1.57)^*^	1.05 (0.88–1.26)

^†^Adjusted for biological, social, and psychological factors associated with stress, depressive symptoms, suicidal ideation in adolescents.

^*^p < 0.05.

Q1, first quantile (reference); Q2, second quantile; Q3, third quantile.

Compared to Q1 of fast-food consumption (reference), Q2 of fast-food consumption was associated with increased depressive symptoms (AOR = 1.16, 95% CI = 1.08–1.25), as was Q3 of fast-food consumption (AOR = 1.38, 95% CI = 1.21–1.57) compared to the reference (Table 3).

### 3.3 Effects of combining consumption of SSBs and fast foods on mental health in adolescents

Combining Q1 of SSB consumption and Q3 of fast-food consumption was associated with increased depressive symptoms (AOR = 1.15, 95% CI = 1.01–1.31) compared to combining Q1 of both SSB and fast-food consumption (reference). In addition, combining Q2 of both SSB and fast-food consumption was associated with increased stress (AOR = 1.18, 95% CI = 1.08–1.30) compared to the reference. Combining Q2 of SSB consumption and Q3 of fast-food consumption was also associated with increased stress (AOR = 1.32, 95% CI = 1.07–1.63) and depressive symptoms (AOR = 1.60, 95% CI = 1.29–1.98) compared to the reference (Table 4).

**Table 4:** Effects of combining consumption of sugar-sweetened beverages and fast-foods on mental health in adolescents (*N* = 24,006).

**Combining consumption of sugar-sweetened beverages and fast-foods**		**Stress**	**Depressive symptoms**	**Suicidal ideation**
**Sugar-sweetened beverage consumption**	**Fast-food consumption**	**Adjusted odds ratio (95% confidence interval)** ^†^		
Q1 (low)	Q1 (low)	1.00	1.00	1.00
Q1 (low)	Q2 (medium)	1.03 (0.91–1.16)	1.12 (0.75–1.65)	0.99 (0.83–1.19)
	Q3 (high)	0.91 (0.63–1.32)	1.15 (1.01–1.31)^*^	0.53 (0.27–1.04)
Q2 (medium)	Q1 (low)	1.03 (0.96–1.11)	1.03 (0.95–1.12)	0.99 (0.89–1.11)
	Q2 (medium)	1.18 (1.08–1.30)^*^	1.09 (0.93–1.28)	1.04 (0.90–1.19)
	Q3 (high)	1.32 (1.07–1.63)^*^	1.60 (1.29–1.98)^*^	1.06 (0.79–1.42)
Q3 (high)	Q1 (low)	1.15 (1.03–1.30)^*^	1.20 (1.08–1.34)^*^	1.18 (1.03–1.34)^*^
	Q2 (medium)	1.26 (1.15–1.38)^*^	1.36 (1.20–1.54)^*^	1.23 (1.11–1.36)^*^
	Q3 (high)	1.28 (1.09–1.50)^*^	1.59 (1.33–1.91)^*^	1.32 (1.06–1.65)^*^

^†^Adjusted for biological, social, and psychological factors associated with stress, depressive symptoms, suicidal idea in adolescents.

^*^p < 0.05.

Q1, first quantile; Q2, second quantile; Q3, third quantile.

reference=combining Q1 of sugar-sweetened beverage consumption and Q1 of fast-food consumption.

Combining Q3 of SSB consumption with Q1 and Q2 of fast-food consumption was associated with increased stress, depressive symptoms, and suicidal ideation. Combining Q3 of SSB consumption and Q1 of fast-food consumption was associated with a 1.15-fold increase in stress (95% CI = 1.03–1.30), 1.20-fold increase in depressive symptoms (95% CI = 1.08–1.34), and 1.18-fold increase in suicidal ideation (95% CI = 1.03–1.34) compared to the reference. Combining Q3 of SSB consumption and Q2 of fast-food consumption was associated with a 1.26-fold increase in stress (95% CI = 1.15–1.38), 1.36-fold increase in depressive symptoms (95% CI = 1.20–1.54), and 1.23-fold increase in suicidal ideation (95% CI = 1.11–1.36) compared to the reference. Finally, combining Q3 of both SSB and fast-food consumption was associated with a 1.28-fold increase in stress (95% CI = 1.09–1.50), 1.59-fold increase in depressive symptoms (95% CI = 1.33–1.91), and 1.32-fold increase in suicidal ideation (95% CI = 1.06–1.65) compared to the reference (Table 4).

## Discussion

This study identified the effects of combining the consumption of SSBs and fast foods on stress, depressive symptoms, and suicidal ideation in adolescents in Korea. The results of this study indicate that more than medium consumption of SSBs or fast foods may lead to increased stress, depressive symptoms, and suicidal ideation in adolescents.

Previous studies also reported that frequent SSB and fast-food consumption were associated with increased stress, depressive symptoms, and suicidality in adolescents (12, 32–34). In a previous study of Korean adolescents (32), consumption of SSBs and fast foods more than 3–4 times per week was associated with increased stress. Similarly, Yim et al. (34) reported that SSB consumption was associated with a 1.04-fold increase in stress. In addition, depressive symptoms increased with the consumption of SSBs and fast foods more than 1–2 times a week (32). Furthermore, daily consumption of SSBs and fast foods was associated with a 1.37–1.4 and 1.24–1.50 times increase in the depression of Iranian adolescents, respectively (15). Regarding suicidal behaviors, consumption of SSBs more than thrice a day (13) and more than seven times a week was associated with increased suicide attempts (35). Frequent fast-food consumption was associated with a 1.31-fold increase in suicide attempts among adolescents (13). Thus, SSB and fast-food consumption may significantly affect adolescents' mental health. According to Oliver et al. (36), higher emotional stress tends to have a significant association with increased consumption of sweeter and high-fat foods. Similarly, more severe depression might be associated with increased consumption of sweet and high-fat foods (34, 37, 38). Jacob et al. (13) proposed that SSB and fast-food consumption might promote suicidal ideation with increased vulnerability *via* high emotional stress and depression.

Regarding the relation between eating and emotions, individuals with emotional stress seek coping methods to minimize negative feelings due to tension. Eating may be the most common, simple, and least conscious behavior to cope with stressful situations (39); therefore, stress might induce increased appetite *via* activation of the nervous system (40). Arnow et al. (41) also reported that emotional triggers (negative emotions) could induce eating, regardless of physical hunger. Furthermore, as stress and depression are usually associated with eating (42), emotional eaters consume sweet and high-fat foods more frequently in response to stressors (43). Finally, emotional eating due to negative emotions could worsen mental health status *via* poor psychological wellbeing and emotion regulation (44). Thus, SSB and fast-food consumption and mental health could be interrelated. In this context, along with the decreased consumption of SSBs and fast foods, healthy coping methods to relieve negative emotions are suggested to prevent emotional eating, including frequent and increased consumption of SSBs and fast foods.

Combining more than medium consumption of SSBs and fast foods had a more significant association with increased stress, depressive symptoms, and suicidal ideation than their independent consumption in adolescents. In addition, combining high consumption of SSBs and low to high consumption of fast foods may have dose-dependent negative effects on stress, depressive symptoms, and suicidal ideation in adolescents. In a previous study, combining the consumption of caffeinated energy drinks and junk foods was more significantly associated with poor mental health than their independent consumption (14). Xu et al. (12) also reported that 40% of Chinese adolescents' psychological symptoms may be associated with the combined consumption of SSBs and fast foods. Thus, combined consumption of SSBs and fast foods may present more negative dose-dependent effects with synergetic effects. In this context, health education in schools and communities should be provided to adolescents and their parents to restrict SSB and fast-food consumption. In addition, creating a school environment that restricts the sale of SSBs and fast foods, such as limits on installing SSB vending machines and fast-food sales in cafeterias, must be considered. Moreover, health drinks and snacks that can replace SSBs and fast foods should be suggested by healthcare providers in school. According to Rocha et al. (45), school-based nutrition education and feeding program targeting the prohibition of purchasing SSBs and fast foods and offering healthy foods can be a tool to promote a secure school food environment for adolescents' health. Thus, for promoting the mental health of adolescents, the results of this study might provide evidence for developing school-based feeding programs targeting the restriction of SSB and fast-food consumption in schools and communities. In the field of public health, the independent and combined negative effects of SSB and fast-food consumption on mental health should be widely publicized, with policy proposals for decreased consumption to promote both the physical and mental health of adolescents. Accordingly, it is expected that the results of this study will serve as a starting point for social concerns and efforts to limit the consumption of SSBs and fast foods for preventing mental health problems in adolescents.

This study may contribute to strengthening the evidence for developing interventions aimed at decreasing SSB and fast-food consumption in adolescents. Nevertheless, this study had several limitations. First, SSB and fast-food consumption were assessed *via* drinking or eating days, without considering the total amount of consumption. Thus, further studies should evaluate adolescents' total amount of SSB and fast-food consumption from all sources. Second, most variables in the 17^*th*^ KYRBS, 2021, including variables in this study, were assessed using single questions. Thus, further studies should apply systematically developed instruments with sufficient validity and reliability to assess these variables more comprehensively. Third, due to its cross-sectional study design, this study could not identify the causal relationship between independent and outcome variables. Causal relationships among these variables may be verified using a longitudinal study. Fourth, this study involved only Korean high school students as participants; however, eating behaviors may differ with sociocultural background according to country. Thus, future studies should be performed with adolescents from various countries and cultures to draw relevant comparisons.

## Conclusion

This study identified the effects of combining consumption of SSBs and fast foods on stress, depressive symptoms, and suicidal ideation among adolescents in Korean high schools. Combining more than medium consumption of SSBs and fast foods was associated with greater stress, depressive symptoms, and suicidal ideation than their independent consumption. In addition, high combined consumption of SSBs, with low to high consumption of fast foods, may have dose-dependent negative effects on stress, depressive symptoms, and suicidal ideation in adolescents. Based on the results of this study, healthcare providers in schools and communities should prioritize developing school/community-based feeding programs (for improving nutrition knowledge and secure food environments) and policies targeting the restriction of SSB and fast-food consumption to promote adolescent mental health.

## Data availability statement

The raw data supporting the conclusions of this article will be made available by the authors, without undue reservation.

## Author contributions

JSR planned and carried out the entire research process, including the preparation of the article, data analysis, interpretation of results, and writing the manuscript.
